# Emodin Alleviates Severe Acute Pancreatitis-Associated Acute Lung Injury by Inhibiting the Cold-Inducible RNA-Binding Protein (CIRP)-Mediated Activation of the NLRP3/IL-1*β*/CXCL1 Signaling

**DOI:** 10.3389/fphar.2021.655372

**Published:** 2021-04-23

**Authors:** Qiushi Xu, Mengfei Wang, Haoya Guo, Huanhuan Liu, Guixin Zhang, Caiming Xu, Hailong Chen

**Affiliations:** ^1^ Department of General Surgery, The First Affiliated Hospital of Dalian Medical University, Dalian, China; ^2^ Institute (College) of Integrative Medicine, Dalian Medical University, Dalian, China

**Keywords:** Severe acute pancreatitis, acute lung injury, emodin, CIRP, NLRP3, CXCL1

## Abstract

**Objective:** Severe acute pancreatitis (SAP) can lead to acute lung injury (ALI). This study investigated the therapeutic effect of emodin and its molecular mechanisms in a rat model of SAP-ALI.

**Methods:** Forty male Sprague-Dawley rats were randomly divided into the groups: Control (CON), SAP (SAP), emodin (EMO), and C23 (C23). The latter three groups of rats were induced for SAP-ALI by retrograde injection of 5% sodium taurocholate into the biliary-pancreatic duct and were treated with vehicle, emodin or C23, respectively. One day post induction, their pancreatic and lung injury was assessed by histology and arterial blood gas analysis. *In vitro*, rat alveolar macrophages (NR8383 cells) were treated with recombinant rat CIRP in the presence or absence of TAK242 (a TLR4 inhibitor), C23 or emodin. The CIRP-mediated activation of the NLRP3/IL-1*β*/CXCL1 signaling in rat lungs and NR8383 cells was determined. Similarly, the role of IL-1*β* in the CIRP-induced CXCL1 expression was investigated.

**Results:** Emodin treatment significantly reduced inflammation and tissue damages in the pancreatic and lung tissues in rats with SAP-ALI, accompanied by decreasing serum amylase, CIRP and IL-1β levels and improving lung function. Furthermore, emodin significantly mitigated the SAP-up-regulated CIRP expression in the pancreatic islets and lung tissues, and attenuated the SAP-activated NF-κB signaling, NLRP3 inflammasome formation and CXCL1 expression in lung resident macrophages as well as neutrophil infiltration in the lungs of rats. In addition, treatment with CIRP significantly activated the NF-κB signaling and NLRP3 inflammasome formation and induced IL-1β and CXCL1 expression and pyroptosis in NR8383 cells, which were abrogated by TAK242 and significantly mitigated by C23 or emodin. Moreover, CIRP only induced very lower levels of CXCL1 expression in IL-1β-silencing NR8383 cells and treatment with IL-1β induced CXCL1 expression in NR8383 cells in a dose and time-dependent manner.

**Conclusion:** Emodin may inhibit the CIRP-activated NLRP3/IL-1β/CXCL1signaling to decrease neutrophil infiltration and ameliorate the SAP-ALI in rats.

## Introduction

The incidence of acute pancreatitis (AP) is increasing worldwide due to high prevalence of obesity and related biliary stones (Forsmark et al., 2016). AP is usually divided into mild, moderately severe and severe acute pancreatitis (SAP), dependent on their severity (Banks et al., 2013). SAP accounts for 20% of total AP patients (Papachristou, 2008), and has a high death rate because of its serious complications (Bai et al., 2007). SAP is usually complicated by acute lung injury (ALI), which can further progress into acute respiratory distress syndrome (ARDS) (Shields et al., 2002). ARDS is the leading cause of SAP- related death (Guo et al., 2016). However, there currently is no effective therapies for decreasing SAP-ALI mortality. Therefore, it is urgent to further explore the pathogenesis of SAP-ALI and new therapeutic strategies.

Cold-inducible RNA-binding protein (CIRP) is an inflammatory mediator and one of the damage-associated molecular pattern molecules (DAMPs). High levels of serum CIRP are detected in patients with hemorrhagic shock or sepsis, and associated with a high mortality rate (Qiang et al., 2013). Functionally, CIRP can deteriorate inflammatory diseases and damage tissues by binding to NOD-like receptors (NLRs) to activate the NLRP3 inflammasome (Bortolotti et al., 2018; Aziz et al., 2019). The NLRP3 inflammasome is a protein complex, which can directly interact with the adaptor apoptosis-associated speck-like protein containing caspase-recruitment domain (ASC) to activate caspase-1, and the activated caspase-1 splits gasdermin D (GSDMD), pro-IL-1*β* and pro-IL-18, leading to cell pyroptosis, IL-1*β* and IL-18 secretion (Chae et al., 2011; Shi et al., 2015). Pyroptosis is a novel form of programmed cell death involved in inflammation to deteriorate the disease process (Kovacs and Miao, 2017). Lung resident macrophages, such as alveolar macrophages (AMs), are key factors for the pathogenesis of ALI/ARDS (Soni et al., 2016; Huang et al., 2018). The pyroptosis of AMs will aggravate the inflammation in the lung by producing inflammatory cytokines, such as IL-1β, IL-18, and others (He et al., 2016; Fan and Fan, 2018). However, little is known on how CIRP regulates AM pyroptosis and inflammatory cytokine production.

Emodin (1,3,8-trihydroxy-6-methylanthraquinone) is a natural ingredient of Rhei Radix et Rhizoma that is a Chinese herbal medicine and has been used for treatment of patients with pancreatitis in China for a long time. Current evidence indicates that emodin has a wide range of pharmacological properties, including immunosuppressive, anti-inflammatory, antioxidant, anti-fibrotic, and antimicrobial activities (Dong et al., 2016; Zhao et al., 2018). Emodin can significantly ameliorate SAP-ALI by inhibiting neutrophil proteases activity (Xu et al., 2020). However, the molecular mechanisms underlying the action of emodin in inhibiting SAP-ALI have not been clarified. Hence, investigation of the molecular mechanisms and potential targets of emodin will be crucial for the development of effective treatment for SAP-ALI.

To explore the molecular mechanisms underlying the action of emodin in treating SAP-ALI and the role of CIRP in the pathogenic process of SAP-ALI, we employed a rat model of SAP-ALI and tested the therapeutic effect of emodin and C23. The C23 is an oligopeptide derived from the CIRP protein (residues 111–125: GRGFSRGGGDRGYGG) and can act as an antagonist by binding to the CIRP receptor with a high affinity (McGinn et al., 2018; Qiang et al., 2013; Zhang et al., 2018). Furthermore, we examined the effect of C23, emodin or TAK242 (ethyl (6R)-6-[N-(2-chloro-4-fluorophenyl)sulfamoyl]cyclohex-1-ene-1-carboxylate*,* a small molecule specific inhibitor of the Toll-like receptor (TLR) 4 signaling) on the CIRP-induced pyroptosis and inflammatory cytokine production in rat AMs *in vitro*. The results indicated that SAP-ALI significantly increased serum CIRP levels, enhanced CIRP expression in the pancreatic islets and lungs, activated the NLRP3 inflammasome, and increased CXCL1 expression and neutrophil infiltration in the lung of rats, which were significantly mitigated by emodin treatment. Moreover, CIRP up-regulated CXCL1 expression in AMs by activating the NLRP3/IL-1*β* pathway *in vitro*, which were abrogated by emodin treatment. Our findings uncover that CIRP is an endogenous pro-inflammatory mediator, contributing to the pathogenesis of SAP-ALI in rats and emodin targets the CIRP-activated NLRP3/IL-1*β* pathway to mitigate the CIRP-induced CXCL1 production in AMs.

## Materials and Methods

### Reagents and Antibodies

The special reagents included emodin and sodium taurocholate (Solarbio Science Technology, Beijing, China), rat CIRP enzyme-linked immunosorbent assay (ELISA) kit (cat#OM546910), anti-ASC (cat#OM204428), anti-CXCL1 (cat#OM248569, Omnimabs, Poway, CA, United States), amylase and IL-1*β* ELISA kits (Shanghai Lengton Biotech, Shanghai, China), antibodies against Ly6G (cat#GTX40912, Genetex, Dallas, TX, United States), CIRP (cat#A6080), p-P65 (cat#AP0123), P65 (cat#A16271), IKB*α* (cat#A16929), p-IKB*α* (cat#AP0707), NLRP3 (cat#A14223), Caspase-1 (cat#A0964), GSDMD (cat#A18281), IL-1*β* (cat#A11369), *β*-actin (cat#AC026 Abclonal Biotech, Wuhan, China), TAK242 and recombinant rat IL-1β (Absin Bioscience, Shanghai, China), HRP-conjugated goat anti-rabbit IgG (H + L) (cat#31460, Thermo, Waltham, MA, United States), mouse anti-F4/80 antibody (cat# SC-52664, Santa Cruz Biotechnology, Santa, Cruz, CA, United States), rabbit anti-glucagon antibody (cat#GB11097), mouse anti-insulin antibody (cat#GB13121), Cy3-conjugated goat anti-rabbit IgG (H + L) (cat#GB21303), Cy5-conjugated goat anti-mouse IgG (H + L) (cat#GB27301), FITC-conjugated goat anti-mouse IgG (H + L) (cat#GB22301, Servicebio Technology, Wuhan, China). C23 and recombinant rat CIRP (Genscript Biotech, Nanjing, China). Others were RNA extraction reagent, RNAex Pro Reagent (Accurate Biotechnology, Changsha, China), the reverse transcription kit and quantitative real-time PCR kit (Vazyme Biotech, Nanjing, China), CIRP, NLRP3, CXCL1, and IL-1*β* primers (Synbio Technologies, Suzhou, China).

### Establishment of a SAP-ALI Rat Models

All experiments were performed in accordance with the experimental protocol approved by the Committee for Research and Animal Ethics of Dalian Medical University (Dalian, China). Male Sprague-Dawley rats, 180–220 g, were obtained from the Experimental Animal Center of Dalian Medical University. The rats were housed in a specific pathogen-free room with consistent temperature of 20–22°C and a cycle of 12 h light-dark and allowed free access to standard rodent chow and water.

The rat model of SAP-ALI was established as descripted previously (Xu et al., 2020). Briefly, 40 rats were randomly divided into the groups: the Control (CON), SAP (SAP), C23 (C23), and emodin (EMO) groups, respectively, (n = 10 per group). The experimental rats were retrograde-infused with 5.0% sodium taurocholate (0.1 ml/100 g body weight, 0.1 ml/min) into their biliopancreatic duct while the CON group of rats received the equivalent volume of 0.9% sterile saline. 2 h later, the C23 and EMO groups of rats were treated with C23 (8 mg/kg body weight, intravenous injection) and emodin (40 mg/kg body weight, by gavage) (Xu et al., 2021), respectively. The EMO group of rats were repeated the same dose of emodin at 12 h post-operation. 24 h after the injection with sodium taurocholate, all rats were anesthetized with 1% pentobarbital sodium (4 mg/100 g bodyweight, Merck KGaA, Darmstadt, Germany), and their arterial blood samples were collected from their abdominal aorta. Their pancreatic and lung tissues were dissected and one part of the tissues were frozen in liquid nitrogen, and then stored at −80°C. The remaining pancreatic and lung tissues were fixed in 10% formalin and embedded in paraffin. Some arterial blood samples were used for arterial blood gas analysis, and the remaining blood samples were centrifuged to prepare serum samples.

### Measurement of Serum CIRP, Amylase, IL-1*β* Levels and Arterial Blood Gas

The levels of serum CIRP, amylase and IL-1*β* in individual rats were analyzed by ELISA using specific kits, according to the manufacturer’s instructions. The blood gas analysis was performed in an automatic blood gas analyzer (RapidPoint 500, Siemens, Berlin and Munich, Germany).

### Histology and Immunohistochemistry

The paraffin-embedded pancreatic and lung tissue sections (5 μm) were routine-stained with hematoxylin and eosin (HE). The pathological changes in each section was scored as 0 to 4 for the degree of acinar necrosis, inflammation, hemorrhage, and edema in the pancreas (Rongione et al., 1997), and as 0–3 for the degrees of edema, leukocyte infiltration, and hemorrhage in the lung in a blinded manner (Osman et al., 1998).

The levels of CIRP and Ly6G expression in pancreatic and lung tissue sections were determined by immunohistochemistry using antibodies against CIRP (1:600) and ly6G (1:500) as described previously (Ramos-Vara, 2017).

### Cell Culture

Rat alveolar macrophage NR8383 cells, were obtained from the Zhong Qiao Xin Zhou Biotechnology (Shanghai, China) and cultured in Ham’s F-12K medium (Thermo) supplemented with 15% fetal bovine serum (FBS) in a humidified atmosphere of 5% CO_2_ at 37°C.

### Cellular Experiments

To determine the effect of CIRP, NR8383 cells were treated with vehicle as the Control or 1.5 μg/ml recombinant rat CIRP for 6 h as the CIRP group. NR8383 cells were pre-treated with TAK-242 (500 µM) for 24 h (Wu et al., 2018) and treated with 1.5 μg/ml CIRP for 6 h. In addition, NR8383 cells were pre-treated with 300 ng/ml C23 for 1 h (Zhang et al., 2018) and treated with 1.5 μg/ml CIRP for 6 h as the CIRP + C23 group. Moreover, NR8383 cells were treated with 1.5 μg/ml CIRP and 20 µM emodin for 6 h as the CIRP + EMO group (Xiang et al., 2017a). To examine the role of IL-1*β* in the CIRP-increased CXCL1 expression in AMs, NR8383 cells were treated with vehicle as the Control or 1.5 μg/ml CIRP for 6 h as the CIRP group. Furthermore, NR8383 cells were transfected with control siRNA or IL-1*β*-specific siRNA (Table 1) for 48 h and treated with 1.5 μg/ml CIRP for 6 h as the NC or Si-IL-1*β* group, respectively. To determine the effect of IL-1*β* on CXCL1 expression, NR8383 cells were treated with various concentrations (0, 1 ng/ml, 5 ng/ml, 10 ng/ml) of recombinant rat IL-1*β* for 24 h or treated with 10 ng/ml IL-1*β* for varying time periods (0, 6, 12, 24 h), respectively.

**TABLE 1:** The sequence of siRNAs.

Gene	Sense	Antisense
IL-1*β*	GCU​UCC​AAG​CCC​UUG​ACU​UTT	AAG​UCA​AGG​GCU​UGG​AAG​CAA

### Transfection

NR8383 cells were transfected with control siRNA or IL-1*β* specific siRNA (Keygen, Nanjing, China, Table 1) using the Lipofectamine TM 2000 (Invitrogen, Carlsbad, CA, United States), according to the manufacturer’s instruction.

### Quantitative Real-Time PCR

Total RNAs were extracted from rat pancreas, lung samples and the different groups of NR8383 cells using the RNAex Pro Reagent (AG), and the resulting RNA samples (1 µg each) were transcribed reversely into cDNA using the HiScript® II Reverse transcriptase (Vazyme). The relative levels of target gene mRNA transcripts to the control GAPDH were quantified by qPCR using the ChamQ universal SYBR® qPCR Master Mix (Vazyme) and specific primers (Table 2). The data were analyzed by the 2^−ΔΔCT^ method.

**TABLE 2:** The sequences of primers.

Genes	Forward primer (5′-3′)	Reverse primer (5′-3′)
GAPDH	CTG​GAG​AAA​CCT​GCC​AAG​TAT​G	GGT​GGA​AGA​ATG​GGA​GTT​GCT
CIRP	GGT​GGT​GGT​AAA​GGA​CAG​G	CCA​TCC​ACA​GAC​TTC​CCA​TTC
NLRP3	GTG​GAC​CTC​AAC​AGA​CGC​TAC​A	GGC​TCC​AAG​TGT​TCA​TCC​TCA
IL-1*β*	GGC​AAC​TGT​CCC​TGA​ACT​CAA	GCT​TCT​CCA​CAG​CCA​CAA​TGA
CXCL1	CAG​ACA​GTG​GCA​GGG​ATT​CA	TGG​GGA​CAC​CCT​TTA​GCA​TC

### Western Blot Analysis

Fresh pancreatic and lung tissues from individual rats were homogenized and lyzed in RIPA lysis buffer (Keygen). Similarly, the different groups of NR8383 cells were lyzed in RIPA lysis buffer. After quantification of protein concentrations, individual lysate samples were separated by sodium dodecyl sulfate polyacrylamide gel electrophoresis (SDS-PAGE) on 10–15% gels (Solarbio, Beijing, China.) and transferred onto polyvinylidene difluoride (PVDF) membranes. The membranes were blocked with 5% fat-free dry milk in TBST for 1 h and incubated with primary antibodies, including anti-CIRP (1:1000), anti-NLRP3 (1:1000), anti-ASC (1:300), anti-caspase-1 (1:1000), anti-IL-1*β* (1:1000), anti-GSDMD (1:1000), anti-p-P65 (1:1000), anti-P65 (1:1000), anti-IKB*α* (1:1000), anti-p-IKB*α* (1:1000), anti-CXCL1 (1:500), and anti-*β*-actin (1:20,000) at 4°C overnight. After being washed, the bound antibodies were detected with goat anti-rabbit IgG (H + L) secondary antibody (1:5000) and visualized using the enhanced chemiluminescent reagent (Keygen). The bands were imaged using the Tanon 5200 imaging system. The relative levels of the target protein to the control *β*-actin were quantified by densitometric analysis using the Image Pro Plus 5.1 software.

### Immunofluorescence

The distributions of insulin, glucagon and CIRP expression in the pancreatic tissue sections were characterized using the tyramide signal amplification (TSA) method (Francisco-Cruz et al., 2020). Briefly, the pancreatic tissue sections were incubated with rabbit anti-glucagon antibody (1:10,000) overnight at 4°C, and the bound antibodies were detected HRP-conjugated goat anti-rabbit IgG (H + L) (1:500). After being washed, the sections were incubated with FITC-labelled TSA reagents. Subsequently, the sections were probed with mouse anti-insulin antibody (1:300) and rabbit anti-CIRP antibody (1:100) at 4°C overnight. The sections were further incubated with Cy3-conjugated goat anti-rabbit IgG (H + L) (1:300) and Cy5-conjugated goat anti-mouse IgG (H + L) (1:300). The fluorescent signals were scanned using the Pannoramic DESK and the images were captured using the software provided (3DHISTECH, Budapest, Hungary).

In addition, the distribution of F4/80 and NLRP3 or F4/80 and CXCL1 was analyzed by immunofluorescence using two sets of antibodies, as described previously (Mori and Cardiff, 2016). Briefly, the lung tissue sections were incubated with mouse anti-F4/80 antibody (1:100) and rabbit anti-NLRP3 (1:300) or rabbit anti-CXCL1 antibody (1:200) overnight at 4°C. The bound antibodies were probed with FITC-conjugated goat anti-mouse IgG (H + L) (1:200) and Cy3-conjugated goat anti-rabbit IgG (H + L) (1:300). The fluorescent signals were captured using an Olympus BX63 fluorescence microscope (Olympus, Tokyo, Japan) at 200× magnification.

#### Flow Cytometry

The percentages of NR8383 cells undergoing pyroptosis in different groups were measured by flow cytometry using the FAM-FLICA caspase Assay Kit (ImmunoChemistry Technologies, Bloomington, MN, United States), in strict accordance with the manufacturer’s instructions.

#### Statistical Analysis

The data are expressed as means ± standard deviation (SD). The difference among groups was analyzed by one-way ANOVA and post hoc Fisher’s least significant difference (LSD) test using SPSS v25.0 (IBM, Armonk, NY, United States). A *p*-value of <0.05 was considered statistically significant.

## Results

### Treatment With Emodin or C23 Effectively Alleviates the Severity of SAP-ALI in Rats

**FIGURE 1:**
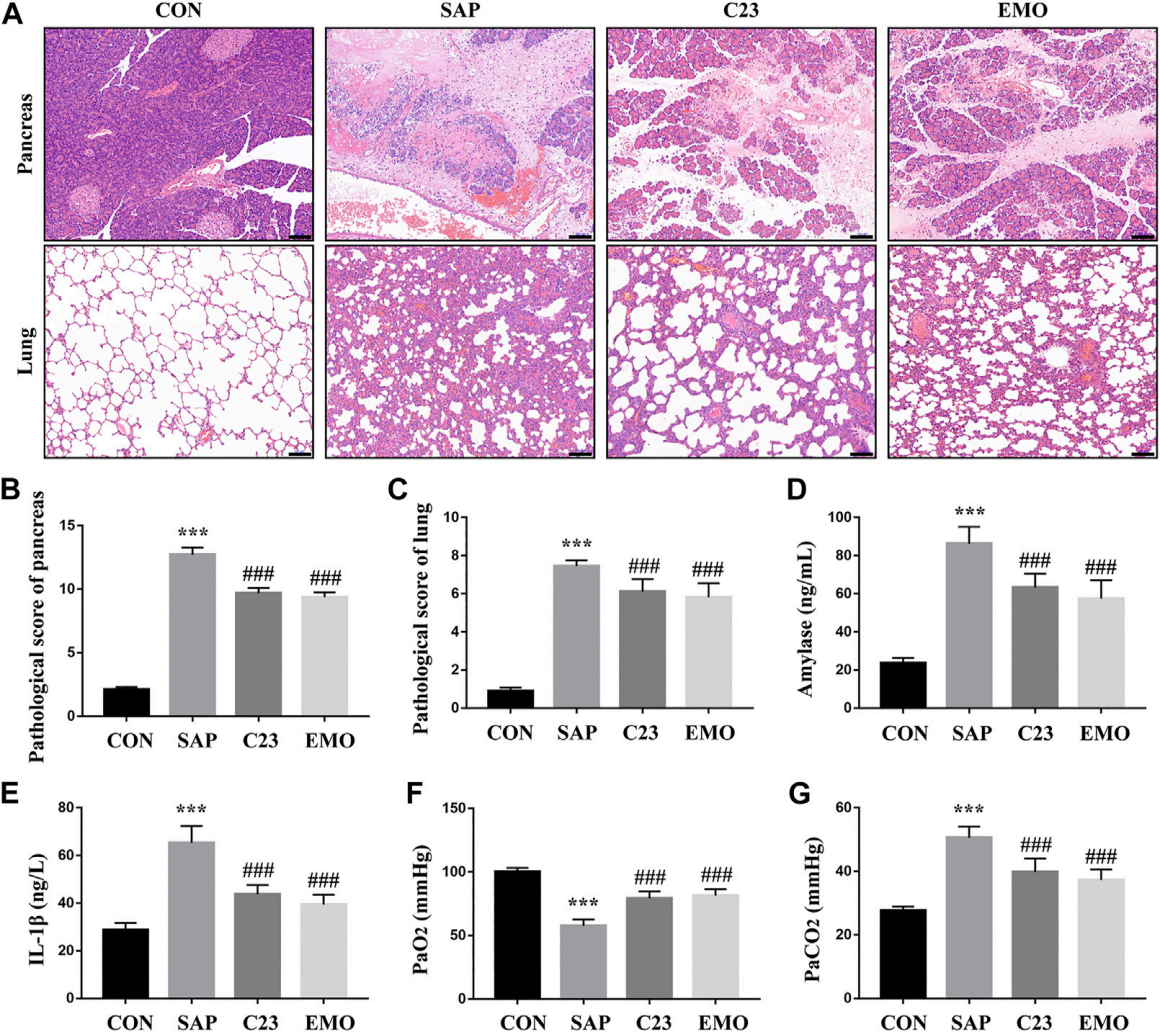
Treatment with emodin or C23 effectively alleviates the severity of SAP-ALI in rats. **(A)** Hematoxylin and Eosin (HE) staining analysis of the pathological changes in the pancreatic and lung tissues (100 × magnification). **(B, C)** Quantitative analysis of pathological scores. **(D)** ELISA analysis of serum amylase levels. **(E)** ELISA analysis of serum IL-1*β* levels. **(F, G)** The analysis of blood arteria gas. The data are representative images or expressed as mean ± SD of each group of rats (*n* = 10 per group) from at least three separate experiments; ^***^ *p* < 0.001 vs. the CON. ^###^ *p* < 0.01 vs. the SAP. Scale bar = 100 μm.

To test the effect of emodin or C23 on the development of SAP-ALI, SD rats were retrograde-infused with sodium taurocholate into their biliopancreatic duct to induce SAP-ALI. 2 h later, the rats were randomized and treated with vehicle (SAP group), emodin or C23. 24 h post induction, the rats were euthanized and their pancreatic and lung tissues were examined by pathology (Figure 1A). While the control rats displayed healthy pancreas and lung tissue morphology, the SAP group of rats exhibited severe edema, inflammation, hemorrhage, and acinar necrosis in their pancreases and edema, leukocyte infiltration and hemorrhage in their lungs, which were less remarkable in the EMO or C23 group of rats. Semi-quantification indicated the histological scores for the pancreas and lung sections in the SAP group were significantly higher than that in the CON group, but were significantly reduced in the EMO or C23 group of rats (P < 0.001 for both, Figures 1B,C). A similar pattern of serum amylase and IL-1*β* levels was detected among the groups of rats, indicating that treatment with emodin or C23 mitigated the SAP and inflammation in rats (Figures 1D,E). Analyses of arterial gas revealed that compared with the CON group, the levels of PaO2 were significantly reduced while the levels of PaCO2 were elevated in the SAP group of rats, which were significantly mitigated in the EMO or C23 group of rats (P < 0.001, Figures 1F,G). Such data indicated that treatment with emodin or C23 significantly mitigated the severity of SAP-ALI in rats.

### Treatment With Emodin Significantly Attenuates the SAP-Induced CIRP Expression in Rats

**FIGURE 2:**
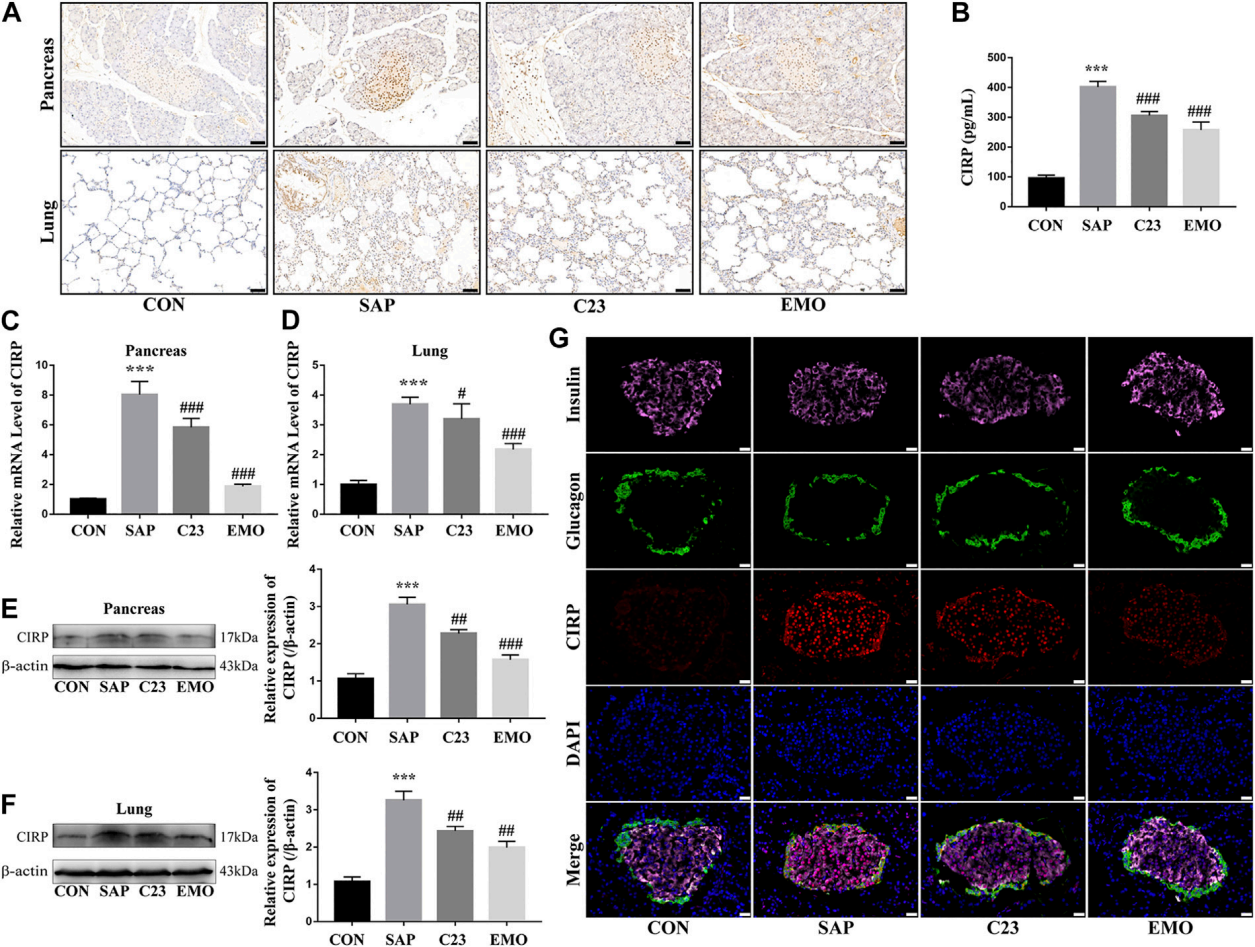
Treatment with emodin significantly attenuates the SAP-induced CIRP expression in rats. **(A)** Immunohistochemistry analysis of CIRP expression in the pancreatic and lung tissues (200 × magnification). Scale bar = 50 μm. **(B)** ELISA analysis of serum CIRP levels. **(C, D)** Quantitative RT-PCR analysis of the relative levels of CIRP to the control GAPDH mRNA transcripts in the pancreatic and lung tissues of rats. **(E, F)** Western blot analysis of the relative levels of CIRP to the control *β*-actin expression in the pancreatic and lung tissues of rats. **(G)** Immunofluorescent analysis of CIRP expression in the pancreatic islets using anti-CIRP (red), anti-insulin (pink), and anti-glucagon (green, 400 × magnification). Scale bar = 20 μm. The data are representative images or expressed as mean ± SD of each group of rats (*n* = 10 per group) from at least three separate experiments; ***p < 0.001 vs. the CON. ^#^ *p* < 0.05, ^##^ *p* < 0.01, ^###^ *p* < 0.001 vs. the SAP.

CIRP is an inflammatory mediator and can bind to the TLR4 to induce pro-inflammatory cytokine production. To understand the molecular mechanisms underlying the action of emodin, we characterized the expression of CIRP in the different groups of rats. As shown in Figure 2A, while there was a little CIPR expression in the pancreatic and lung tissues of the control rats, the levels of CIRP expression significantly increased in the pancreatic islets and lungs of the SAP group of rats. In contrast, the levels of CIRP expression in the pancreatic islets and lung tissues were obviously reduced in the C23 or EMO group of rats, particularly in the EMO group. Furthermore, the levels of serum CIRP in the C23 or EMO group of rats were significantly lower than that in the SAP, but remained higher than that in the CON group of rats (P < 0.001 for all, Figure 2B). A similar pattern of CIRP mRNA transcripts and protein expression was detected in the pancreatic and lung tissues in the different groups of rats (Figures 2C–F). Finally, immunofluorescent analysis revealed that CIRP expression was induced in *β* and *α* cells in the pancreatic islets of the SAP group of rats, which was obviously reduced in the C23 group, particularly in the islet *β* cells and further decreased in the islets of the EMO group of rats (Figure 2G). Collectively, the results demonstrated that treatment with emodin, like C23, significantly mitigated the SAP-up-regulated CIRP expression in the pancreatic islets and lung tissues of rats.

### Treatment With Emodin, Like C23, Significantly Attenuates the SAP-Activated NF-κB Signaling and NLRP3 Inflammasome Formation in the Lungs of Rats

**FIGURE 3:**
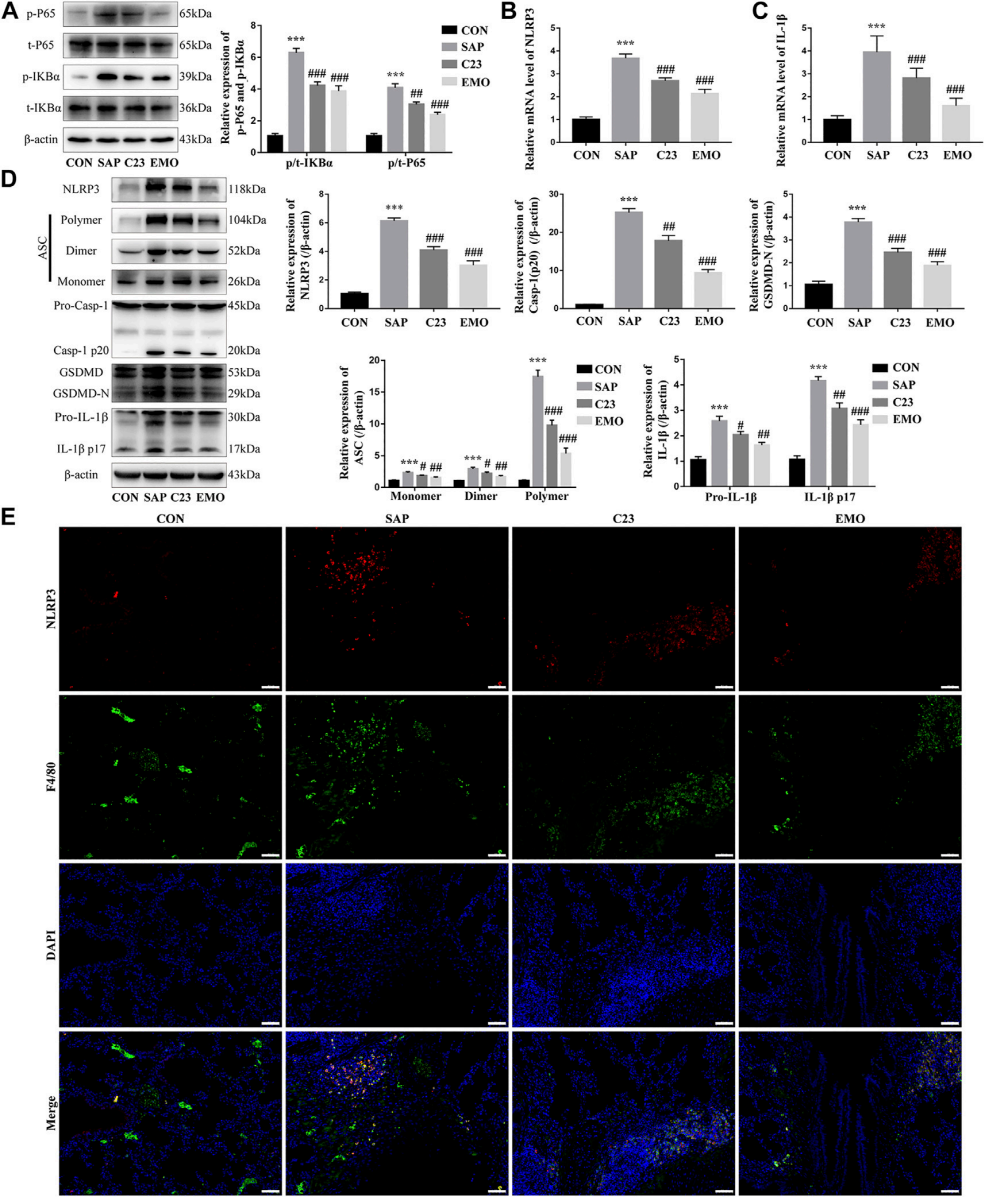
Treatment with emodin, like C23, significantly attenuates the SAP-activated NF-κB signaling and NLRP3 inflammasome formation in the lungs of rats. **(A)** Western blot analysis of the relative levels of NF-κBp65 and IKB*α* to the control *β*-actin expression and phosphorylation in the lung tissues of rats. **(B, C)** Quantitative analysis of the relative levels of NLRP3 and IL-1β to the control GAPDH mRNA transcripts in the lung tissues of rats. **(D)** Western blot analysis of the relative levels of NLRP3, ASC (Monomer, Dimer, Polymer), caspase1 (Pro-Casp-1, Casp-1 p20), GSDMD (GSDMD, GSDMD-N), and IL-1*β* (Pro-IL-1*β*, IL-1*β* p17) to the control *β*-actin expression in the lung tissues of rats. **(E)** Immunofluorescent analysis of the NLRP3 expression in the lung resident macrophages using anti-NLRP3 (red) and anti-F4/80 (green). The data are representative images (200 × magnification) or present as mean ± SD of each group of rats (*n* = 10 per group) from at least three separate experiments; ***p < 0.001 vs. the CON. ^#^ *p* < 0.05, ^##^ *p* < 0.01, ^###^ *p* < 0.001 vs. the SAP. Scale bar = 50 μm.

To further understand the action of emodin, we investigated the effect of emodin on the NF-κB signaling and NLRP3 inflammasome formation in the lungs of the different groups of rats. In comparison with the CON group, significantly higher levels of NF-κBp65 and IKB*α* phosphorylation were detected in the lung of the SAP group of rats, which were significantly reduced in the lungs of the EMO or C23 group of rats, indicating that treatment with emodin or C23 mitigated the SAP-activated NF-κB signaling in the lungs of rats (Figure 3A). A similar pattern of NLRP3 and IL-1*β* mRNA transcripts and protein expression as well as polymer, dimer and monomer ASC, caspase-1, GSDMD-N, pro-IL-1*β*, cleaved IL-1*β* (p17) expression was observed in the lungs of different groups of rats (Figures 3B–D). Immunofluorescent analysis using anti-NLRP3 and anti-F4/80, a marker of resident macrophages (Miyabe et al., 2019) indicated that the NLRP3 expression was mainly overlapped with F4/80 in macrophages in the lungs of the SAP group of rats, which was significantly reduced in the lungs of the EMO or C23 group of rats (Figure 3E). Together, such data indicated that treatment with emodin or C23 significantly mitigated the SAP-activated NF-κB signaling and NLRP3 inflammasome formation in the lungs of rats.

### Treatment With Emodin or C23 Reduces the SAP-Enhanced CXCL1 Expression and Neutrophil Infiltration in the Lungs of Rats

**FIGURE 4:**
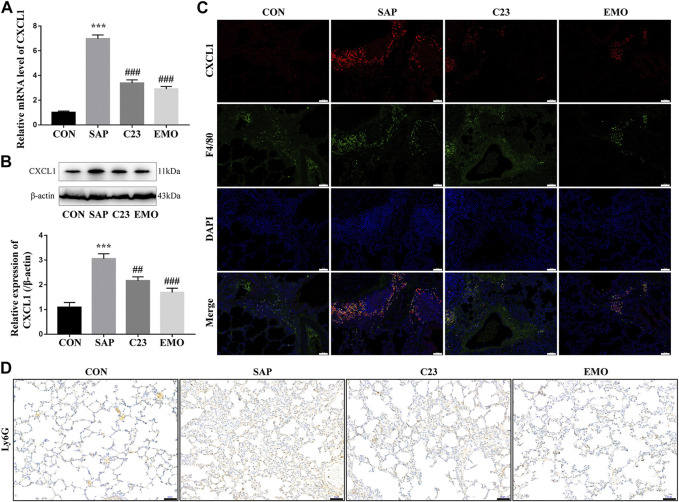
Treatment with emodin or C23 reduces the SAP-enhanced CXCL1 expression and neutrophil infiltration in the lungs of rats. **(A)** Quantitative RT-PCR analysis of the relative levels of CXCL1 to the control GAPDH mRNA transcripts in the lungs of rats. **(B)** Western blot analysis of the relative levels of CXCL1 to the control *β*-actin expression in the lungs of rats. **(C)** Immunofluorescent analysis of CXCL1 expression in lung resident macrophages using anti-CXCL1 (red) and anti-F4/80 (green) (200 × magnification). **(D)** Immunohistochemistry analysis of infiltrated neutrophils in the lung tissues of rats using anti-Ly6G antibodies. The data are representative images (200 × magnification) or expressed as mean ± SD of each group of rats (*n* = 10 per group) from at least three separate experiments; ***p < 0.001 vs. the CON. ^##^ *p* < 0.01, ^###^ *p* < 0.001 vs. the SAP. Scale bar = 50 μm.

CXCL1 is critical for the migration of neutrophils and their infiltration in the lung contributes to the development of ALI (Bhatia et al., 1998; De Filippo et al., 2013; Inoue et al., 1995; Keck et al., 2002). To understand the action of emodin in inhibiting the SAP-ALI, we tested the levels of CXCL1 expression in the lungs of different groups of rats. We found that the levels of CXCL1 mRNA transcripts and protein expression in the lungs of the SAP group were significantly higher than that in the CON group of rats, but were significantly reduced in the EMO or C23 group of rats (Figures 4A,B). Immunofluorescent analysis indicated that the CXCL1 expression was co-localized with F4/80 in the lung tissue sections of the SAP group of rats, but little was detected in the lungs of other groups of rats (Figure 4C). Such data indicated that CXCL1 was mainly expressed by lung resident macrophages during the process of SAP-ALI. Immunohistochemistry analysis using antibodies against Ly6G, a marker of neutrophils (Feng et al., 2017) revealed that there were many Ly6G + neutrophils in the lung and alveolar spaces of the SAP group of rats, which was obviously reduced in the C23 and EMO groups of rats (Figure 4D). Hence, treatment with emodin or C23 significantly reduced the SAP-up-regulated CXCL1 expression and neutrophil infiltration in the lungs of rats.

### Treatment With Emodin, Like C23, Mitigates the CIRP-Activated NF-κB Signaling, NLRP3 Inflammasome Formation, and Pyroptosis in NR8383 Cells

**FIGURE 5:**
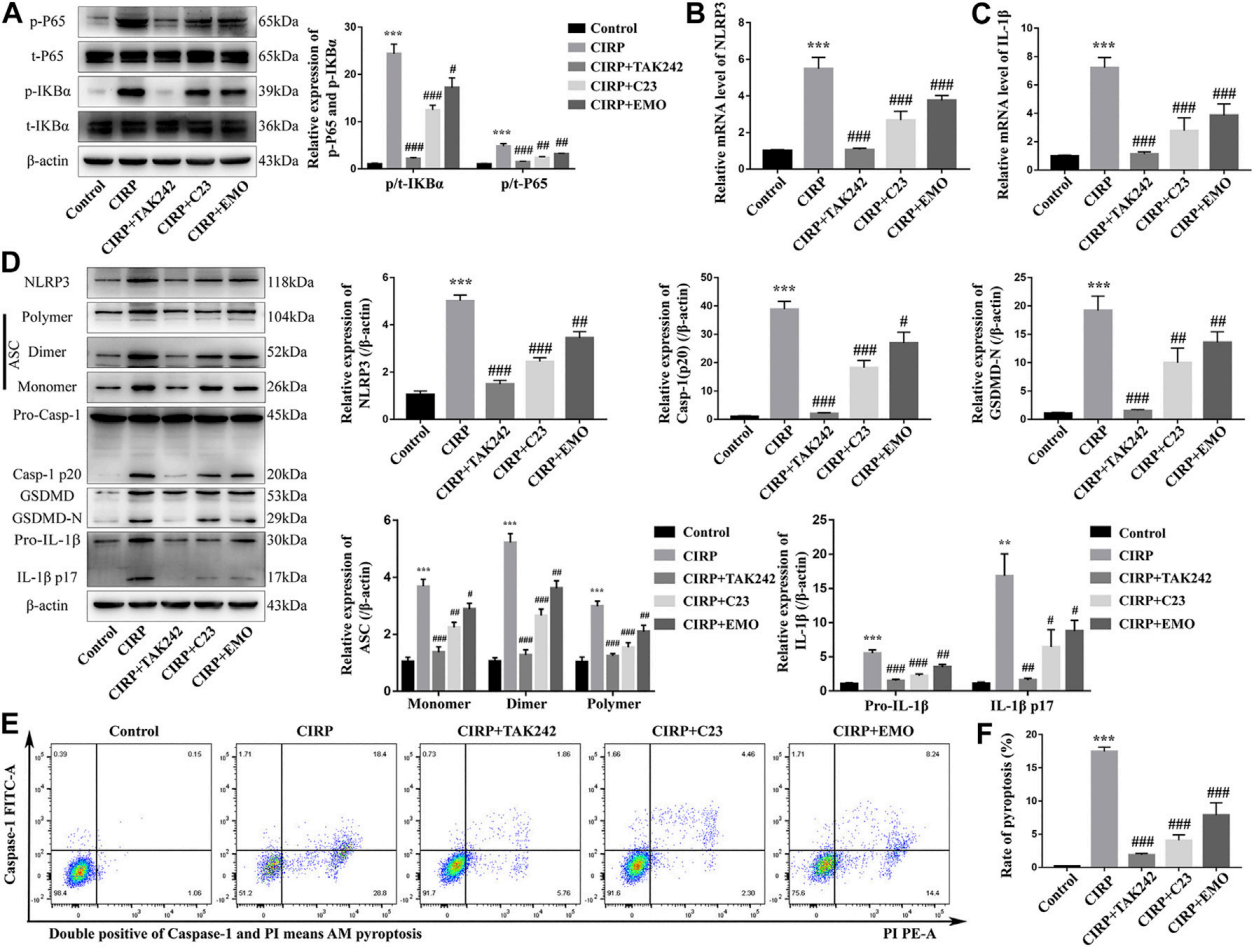
Treatment with emodin, like C23, mitigates the CIRP-activated NF-κB signaling, NLRP3 inflammasome formation, and pyroptosis in NR8383 cells. NR8383 cells were treated with vehicle as the control, or CIRP in the presence of TAK242, emodin or C23 for 6 h. **(A)** Western blot analysis of the relative levels of NF-κBp65 and IKBα to the control *β*-actin expression and their phosphorylation in the different groups of NR8383 cells. **(B, C)** Quantitative RT-PCR analysis of the relative levels of NLRP3 and IL-1*β* to the control GAPDH mRNA transcripts in the different groups of NR8383 cells. **(D)** Western lot analysis of the relative levels of NLRP3, ASC (Monomer, Dimer, Polymer), caspase1 (Pro-Casp-1, Casp-1 p20), GSDMD (GSDMD, GSDMD-N), and IL-1*β* (Pro-IL-1*β*, IL-1*β* p17) to *β*-actin expression in NR8383 cells. **(E, F)** Flow cytometry analysis of the frequency of NR8383 cells undergoing pyroptosis in NR8383 cells. The data are representative images, flow cytometry charts or expressed as mean ± SD of each group of cells from three separate experiments; ***p < 0.001 vs. the Control group. ^#^ *p* < 0.05, ^##^ *p* < 0.01, ^###^ *p* < 0.001 vs. the CIRP group.

To explore the effect of emodin on the CIRP-activated NF-κB signaling, inflammasome formation and pyroptosis in macrophages, NR8383 cells were stimulated with recombinant rat CIRP for 6 h. We found that CIRP stimulation significantly increased the levels of IκBα and NF-κBp65 phosphorylation, a hall mark of the activation of the NF-κB signaling in NR8383 cells, which were nearly completely abrogated by TAK242 treatment and significantly mitigated by C23 or emodin treatment (Figure 5A). A similar pattern of NLRP3 and IL-1β mRNA transcripts and NLRP3, ASC, caspase-1 (p20), GSDMD-N, and IL-1β protein expression was observed in the different groups of NR8383 cells (Figures 5B–D). Flow cytometry analysis revealed that CIRP triggered a high frequency of caspase-1+PI + NR8383 cells, a hallmark of cell pyroptosis, which was also completely abrogated by treatment with TAK242, and significantly reduced by treatment with C23 or emodin (Figures 5E,F). Thus, treatment with emodin significantly reduced the CIRP-activated NF-κB signaling, NLRP3 inflammasome formation, and pyroptosis in NR8383 cells *in vitro*.

### Treatment With Emodin Mitigates the CIRP-Induced IL-1*β*-dependent CXCL1 Expression in NR8383 Cells

**FIGURE 6:**
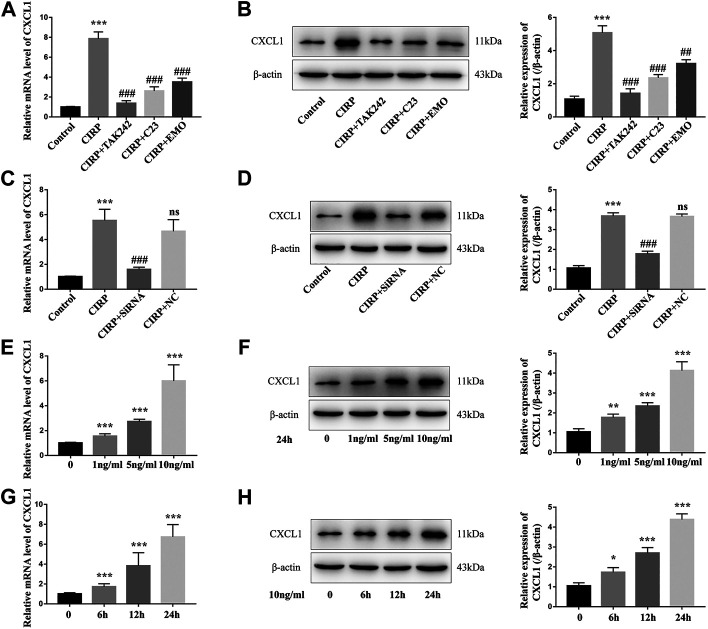
Treatment with emodin mitigates the CIRP-induced IL-1*β*-dependent CXCL1 expression in NR8383 cells. **(A)** Quantitative RT-PCR analysis of the relative levels of CXCL1 to the control GAPDH mRNA transcripts in NR8383 cells. **(B)** Western blot analysis of the relative levels of CXCL1 to the control *β*-actin expression in NR8383 cells. **(C and D)** CIRP only induced very lower levels of CXCL1 expression in the IL-1*β* silencing NR8383 cells. **(E–H)** IL-1β enhanced CXCL1 expression in NR8383 cells in a dose- and time-dependent manner. The data are representative images or expressed as mean ± SD of each group of cells from three separate experiments; ^*^ *p* < 0.05, **p < 0.01, ***p < 0.001 vs. the Control group. ^##^ *p* < 0.01, ^###^ *p* < 0.001 vs. the CIRP group. NS, not significant.

Finally, we tested whether CIRP could modulate CXCL1 expression and how treatment with emodin, or C23 could alter its effect in NR8383 cells. First, we found that treatment with CIRP significantly up-regulated CXCL1 mRNA transcripts and protein expression in NR8383 cells, which was abrogated by TAM242 treatment, and significantly mitigated by C23 or emodin treatment (Figures 6A,B). Because CIRP induced the NLRP3 inflammasome formation and CXCL1 and IL-1*β* expression in NR8383 cells, we tested whether IL-1*β* was crucial for CIRP to induce CXCL1 expression in NR8383 cells. We found that CIRP only induced very lower levels of CXCL1 expression in IL-1*β* silencing NR8383 cells, relative to that in the control siRNA-transfected NR8383 cells (Figures 6C,D). Furthermore, treatment with different concentrations of IL-1*β* for varying periods revealed that IL-1*β* induced CXCL1 expression in NR8383 cells in a dose- and time-dependent manner (Figures 6E–H). Therefore, treatment with emodin mitigates the CIRP-induced IL-1*β*-dependent CXCL1 expression in NR8383 cells *in vitro*.

## Discussion

SAP can cause multiple organ dysfunction syndrome, commonly affecting the lung to induce ALI, even to ARDS, which is responsible for 60–70% of SAP-related deaths (Guice et al., 1988; Johnson and Abu-Hilal, 2004; Mofidi et al., 2006; Banks et al., 2013; Schepers et al., 2019). CIRP is an inflammatory mediator and can deteriorate inflammation and tissue damages in the inflamed pancreas (Linders et al., 2020) and can induce ALI (Cen et al., 2017; Yang et al., 2016; Zhang et al., 2020). Actually, the levels of serum CIRP are associated with the severity and prognosis of SAP (Gong et al., 2017). In this study, we investigated the potential role of CIRP in the pathogenesis of SAP-ALI and the effect of emodin treatment on the severity of SAP-ALI and potential mechanisms in a rat model of SAP-ALI. We found that while induction of SAP caused both pancreatic and lung tissue damages and impaired the lung function, accompanied by elevating serum IL-1*β*, treatment with emodin or C23 significantly mitigated the severity of SAP-ALI and improved lung function as well as decreased serum IL-1*β* in rats. Such findings were consistent with previous observations that emodin reduces the mortality of SAP (Xiang et al., 2017b) and ameliorates SAP-ALI (Xu et al., 2020). Thus, emodin may be a promising candidate for intervention of SAP-ALI.

**FIGURE 7:**
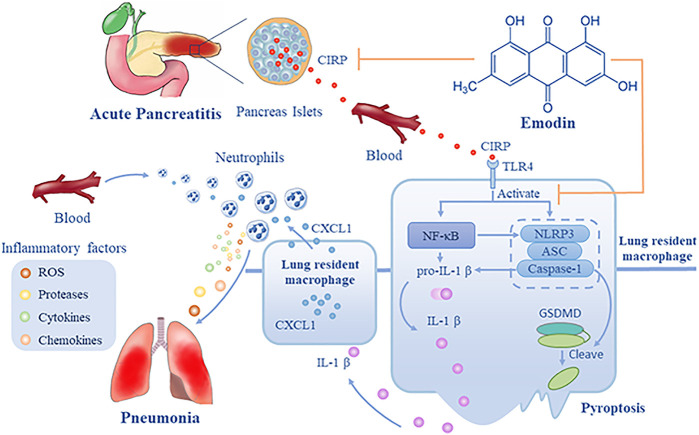
A diagram illustrates the potential mechanisms underlying the action of emodin in inhibiting SAP-ALI. During the process of SAP, CIRP is released from pancreatic islets into the blood and circulates into the lung, leading to the activation of the NF-κB signaling and NLRP3 inflammasome formation and pyroptosis of lung resident macrophages that release IL-1β. Subsequently, IL-1β induces CXCL1 expression in macrophages and others, and the increased CXCL1 recruits neutrophil infiltration into the lung, where the infiltrated neutrophils produce ROS, proteases, cytokines, and chemokines, damaging the lung tissues. Emodin may inhibit CIRP expression and the CIRP-related NF-kB signaling and NLRP3 inflammasome formation, IL-1*β*, and CXCL1 expression, mitigating neutrophil infiltration and lung damages, alleviating SAP-ALI. Hence, emodin targets the CIRP/NLRP3/IL-1β/CXCL1 pathway, the critical players in the pathogenesis of SAP-ALI, and may be a promising candidate for intervention of SAP-ALI.

Because CIRP is a critical factor for ALI, we explored the effect of emodin treatment on CIPR expression in both the lung and pancreas of rats. We found that induction of SAP significantly up-regulated CIRP expression in the pancreatic islets and lung tissues of rats, accompanied by increased levels of serum CIRP. Such data extended previous findings that up-regulated CIRP expression is detected during the process of shock (Qiang et al., 2013) and acute hypoxia (Zhou et al., 2014). It is possible that SAP may cause hypovolemia, hypotension and microvascular thrombosis and lead to tissue hypoperfusion and hypoxia that up-regulate CIRP expression during the process of SAP (Garg and Singh, 2019). Interestingly, we detected inducible CIRP expression in *β* and *α*-cells of the pancreatic islets, which may explain high levels of serum CIRP as *β* and *α*-cells can secrete insulin and glucagon into blood circulation. More importantly, we found that treatment with emodin or C23 significantly mitigated the SAP-up-regulated CIRP expression in the pancreatic islets and lung tissues, which may contribute to their therapeutic effect on inhibiting the severity of SAP-ALI in rats (Figure 7).

CIRP can bind to the TLR4 to activate the nuclear factor κB (NF-κB) signaling (Chen et al., 2016; Zhou et al., 2020), which induces the expression of NLRP3, pro-IL-1*β* and many other cytokines, leading to activation of NLRP3 inflammasome (Schroder and Tschopp, 2010; Wang et al., 2018; Yu et al., 2017). The activation of NLRP3 inflammasome will deteriorate inflammation of pancreatitis (Hou et al., 2019; Sendler et al., 2020), and esults in production of IL-1*β*, IL-18 (Fan and Fan, 2018), and HMGB1 (Hou et al., 2018), which are associated with systemic injury. In this study, we found that treatment with emodin, like C23, significantly mitigated the SAP-activated NF-κB signaling and NLRP3 inflammasome formation as well as IL-1*β*, CXCL1 expression, reducing neutrophil infiltration in the lungs of rats (Figure 7). Similar effects of emodin on the CIRP-activated NF-κB signaling and NLRP3 inflammasome formation, IL-1*β*, CXCL1 expression were observed in NR8383 cells *in vitro*. Previous studies have shown that emodin can inhibit the NF-κB activation and subsequent inflammatory responses and the activation of NF-κB can promote the activation of NLRP3 inflammasome (Bauernfeind et al., 2009; Xiao et al., 2014; Ding et al., 2018a). These, together with the effects of emodin, similar to C23, suggest that emodin may inhibit the activation of NLRP3 inflammasome by inhibiting the CIRP-induced NF-κB activation in resident macrophages. We are interested in investigating how emodin inhibits the NF-κB activation and NLRP3 inflammasome formation during the process of SAP-ALI.

CXCL1 is critical for the migration of neutrophils into the lung while infiltrated neutrophils can produce reactive oxygen species (ROS), proteases, cytokines, and chemokines, deteriorating inflammation and lung damages (De Filippo et al., 2013; Thieblemont et al., 2016). The significantly decreased CXCL1 expression in macrophages by eomdin treatment may be a key step to inhibit SAP-ALI, because macrophages can mediate and amplify the inflammatory cascade during the pathological progress of SAP by secreting CCL2, CXCL1, IL-1*β*, and other pro-inflammatory mediators (Shamoon et al., 2016; Jiménez-Alesanco et al., 2019). Apparently, SAP induced CIRP expression that activated the NF-κB signaling and the NLRP3 inflammasome, increasing IL-1*β* and CXCL1 expression in the lung to promote neutrophil infiltration, damaging the lung tissues. Interestingly, we found that treatment with TAK242 abrogated the CIRP-induced biological effects on NR8383 cells, indicating that CIRP through the TLR4 activated the NF-κB signaling and NLRP3 inflammasome formation in macrophages, consistent with previous reports (Qiang et al., 2013; Li et al., 2017; Bolognese et al., 2018). In addition, we found that treatment with IL-1*β* up-regulated CXCL1 expression in NR8383 cells in a dose and time-dependent manner. This, together with the fact that CIRP only induced very lower levels of CXCL1 expression in the IL-1*β* silencing NR8383 cells, indicated that CIRP promoted the production of CXCL1 in macrophages by activating the NLRP3/IL-1*β* pathway. It is like that IL-1*β* through its receptor of IL-1RI activates the downstream NF-κB and AMPK signaling to induce CXCL1 expression in macrophages and other types of cells.

In the present study, we demonstrated that CIRP expression was significantly up-regulated in both *α* and *β*-cells of the pancreatic islets, which was significantly mitigated by emodin treatment, accompanied by decreasing serum CIRP in rats. Our novel data indicated that emodin ameliorated the severity of SAP-ALI by through inhibiting the CIRP-related signaling and decreased neutrophil infiltration in the lung of rats by attenuating the CIRP-induced IL-1*β*-dependent CXCL1 expression. A previous study has showed that emodin can relieve SAP-associated lung injury by inhibiting the activation of NLRP3 inflammasome (Gao et al., 2020). Our study also indicated that emodin reduced cell pyroptosis and IL-1*β* production as well as CXCL1 expression by inhibiting the activation of NLRP3 inflammasome induced by CIRP, which may be the key mechanism underlying the action of emodin in reducing SAP-ALI severity in rats. Treatment with emodin, like C23, can inhibited the CIRP-induced biological process in this model, which extended previous findings that emodin inhibited the TLR4 expression during the process of SAP and other inflammatory diseases (Li et al., 2009; Ding et al., 2018b). Accordingly, we speculate that emodin may act as an inhibitor of the CIRP-TLR4 signaling. However, the molecular mechanisms by which emodin decreases CIRP expression and the CIRP-TLR4 signaling during the process of SAP-ALI remain to be explored.

In conclusion, our data indicated that emodin treatment significantly mitigated the severity of SAP-ALI and IL-1*β* expression, and improved the lung function in rats. Mechanistically, emodin significantly decreased the SAP-up-regulated CIRP expression in the pancreatic islet and lung tissues as well as the CIRP content in serum to attenuate the CIRP-activated NF-κB signaling, NLRP3 inflammasome formation, IL-1*β*, and CXCL1 expression to reduce neutrophil infiltration in the lungs of rats. Such novel data indicate that CIRP, as a key inflammatory mediator, contributes to the pathogenesis of SAP-ALI by promoting neutrophil infiltration in the lung through activating the NF-κB and NLRP3/IL-1β/CXCL1 pathways. Thus, our findings may provide new insights into the role of CIRP in the pathogenesis of SAP-ALI and the pharmacological action of emodin in inhibiting inflammation during the process of SAP-ALI. Therefore, emodin may be a promising candidate for intervention of SAP-ALI and other inflammatory diseases.

## Data Availability

The original contributions presented in the study are included in the article/Supplementary Material, further inquiries can be directed to the corresponding authors.
